# Dengue forecasting in São Paulo city with generalized additive models, artificial neural networks and seasonal autoregressive integrated moving average models

**DOI:** 10.1371/journal.pone.0195065

**Published:** 2018-04-02

**Authors:** Oswaldo Santos Baquero, Lidia Maria Reis Santana, Francisco Chiaravalloti-Neto

**Affiliations:** 1 Department of Preventive Veterinary Medicine and Animal Health, School of Veterinary Medicine, University of São Paulo, São Paulo, Brazil; 2 Department of Epidemiology, School of Public Health, University of São Paulo, São Paulo, Brazil; 3 Epidemiological Surveillance Center, Department of Health, São Paulo State, São Paulo, Brazil; Columbia University, UNITED STATES

## Abstract

Globally, the number of dengue cases has been on the increase since 1990 and this trend has also been found in Brazil and its most populated city—São Paulo. Surveillance systems based on predictions allow for timely decision making processes, and in turn, timely and efficient interventions to reduce the burden of the disease. We conducted a comparative study of dengue predictions in São Paulo city to test the performance of trained seasonal autoregressive integrated moving average models, generalized additive models and artificial neural networks. We also used a naïve model as a benchmark. A generalized additive model with lags of the number of cases and meteorological variables had the best performance, predicted epidemics of unprecedented magnitude and its performance was 3.16 times higher than the benchmark and 1.47 higher that the next best performing model. The predictive models captured the seasonal patterns but differed in their capacity to anticipate large epidemics and all outperformed the benchmark. In addition to be able to predict epidemics of unprecedented magnitude, the best model had computational advantages, since its training and tuning was straightforward and required seconds or at most few minutes. These are desired characteristics to provide timely results for decision makers. However, it should be noted that predictions are made just one month ahead and this is a limitation that future studies could try to reduce.

## Introduction

Globally, the number of cases has been on the increase since 1990 and this trend has also been found in Brazil and its most populated city—São Paulo [1]. Dengue is one of the most important arthropod-borne viral infection of humans [2]. Its estimated burden is concentrated in Asia and the Americas [3]. In 2015, Brazil accounted for 68% of probable cases in the Americas [4].

Surveillance systems based on predictions allow for timely decision making processes, and in turn, timely and efficient interventions to reduce the burden of the disease [5]. But this only happens when predictions are accurate (do not have systematic errors) and precise (the magnitude of random errors is compatible with the intended application), something that must be evaluated instead of supposed. *Google Flu Trends* [6] is a popular example of both an effort to improve surveillance systems with predictions and of the problems caused by inaccurate predictions [7].

The complexity of dengue dynamics challenges the development of predicting models. However, advances and availability of predictive models, computational capabilities and the incorporation of key predictors have provided necessary tools to tackle this problem. Furthermore, novel data streams and frameworks to use them might also enhance the capability of dengue surveillance systems [8,9].

Meteorological variables influence vector dynamics, agent development, and mosquito/human interactions [10]. Variables such as temperature, precipitation and relative humidity, as well as lagged values of dengue cases, have been reported as key predictors [3,11–13]. As for predictive models, choices adopted in previous studies include seasonal autoregressive integrated moving average models (SARIMA), generalized additive models (GAM), artificial neural networks (ANN) and spatiotemporal Bayesian models [11–18].

The use of complex predictive models is justified if they improve the predictive performance relative to simpler benchmarks. These improvements have been shown for dengue prediction using categorical levels (low, medium, high) [19]. For continuous predictions, a simple benchmark that can be used to compare the performance of complex models is the naïve model, which predict the value of a sequence at time *t* as being equal to the value of that sequence at time *t-1*; despite its simplicity, it performs well for many economic and financial time series [20]. There is little point in adopting a complex model if it does not outperform a simpler benchmark or if its gains in prediction performance are minimal and do not outweigh the costs associated with the forecasting process (data acquisition, implementation, run time and reproducibility).

In recent years, ANN have won contests in pattern recognition and machine learning [21]. Furthermore, the availability of advanced open source libraries and the increasing power of personal computers allow the use of ANN in a wide range of domains. Dengue forecasting is one domain that can exploit the advantages of ANN and indeed, some researches have already started exploring this potential [14,15]. There are many types of ANN, such as the long short-term memory recurrent neural networks (LSTM), which have emerged as an effective and scalable model for several learning problems related to sequential data [22], as is the case of time series.

Since surveillance systems will benefit from a improved knowledge of the relative performance of predictive models, we proposed to carry out a comparative study of dengue prediction in São Paulo city; using the SARIMA, GAM, ANN (including the LSTM) and naïve models.

## Methods

This section is subdivided in twelve subsections. After describing the Data sources and their content, we presented the procedures for Data partition, Exploratory analysis and Predictor selection that preceded model training. Each one of the next five subsections (Naïve model, GAM, ANN, SARIMA, Ensemble model) briefly contextualizes and describes the models. Then, we described the Predictive performance and ended reporting the used Software and presenting the Ethics statement.

### Data

We obtained dengue data from the Epidemiological Surveillance Center of São Paulo State Department of Health (Centro de Vigilância Epidemiológica da Secretaria de Estado da Saúde). Raw data were in files, one per year, from January 2000 to April 2016. Each file had the date (daily resolution), the city and the classification of each notification. We excluded the cases with a “rule out” classification, which according to the Brazilian Ministry of Health, are suspect cases with negative laboratory result (two negative results from paired-IgM samples, properly collected and transported), positive laboratory result for another disease, or without laboratory result but with clinical and epidemiological findings compatible with other disease [23].

Our source of meteorological variables was the National Institute of Meteorology (Instituto Nacional de Meteorologia) [24]. The monthly series also spanned from January 2000 to April 2016 and included temperature (minimum, mean and maximum), precipitation and relative humidity (S1 Fig).

### Data partition

Models can have good predictive performance in the data used to train them but not necessarily in new data. To avoid this issue and find models with good predictive performance in new data, data can be partitioned to use one set to train the models, other to tune model parameters (for parameters not learned from data, find the values that improve predictive performance), and other to test the predictive performance in new data [25]. For tuning of parameters, cross-validation (CV) is a resampling procedure that divides training data to use a subset for model training itself, and the remaining subset to calculate the predictive performance; the procedure is repeated multiple times to obtained a summarized measure of predictive performance [25]. In the case of time series, the procedure must preserve the time order and this implies that the subset used to calculate the predictive performance must be more recent than the subset used for training, This particularity of time series reduces the number of possible resamples, and the more times the procedure is repeated, the smaller the training subset. In our case, the training set comprised the monthly series between January 2000 and December 2014, while the remaining was the test set. The training set was further partitioned to create time series cross-validation (CV) subsets for tuning parameters. Each CV subset, preserving the time order, had a training subset with 165 months and a validation subset with the following 6 months. The first CV subtest started in January 2000, the second in February 2000 and so on, until the seventh, which began in July 2000 and ended in December 2014. We did not use the second semester for validation because it had few cases and small variability. We did not use more subsets to avoid smaller training subsets.

### Exploratory analysis and preprocessing

Two months had missing mean and maximum temperatures, so we imputed them by linear interpolation. The maximum correlation between the number of cases (prediction target) and lagged values—of the number of cases and the meteorological variables—always occurred among the first three lags (Table 1), so we restricted the subsequent training of models to these lags in order to avoid extra computational costs added by variables with small predictive potential.

**Table 1:** Correlation between the number of cases and lags 1–3 of six predictors.

Predictor (lagged)	Lag 1	Lag 2	Lag 3
Cases	0.680	0.240	0.048
Precipitation	0.101	0.208	0.239
Maximum temperature	0.180	0.334	0.374
Mean temperature	0.207	0.335	0.358
Minimum temperature	0.219	0.315	0.310
Relative humidity	0.062	-0.071	-0.101

We standardized all predictors, which were lags (up to third order) of the number of cases and the meteorological variables. For the SARIMA, we also applied a logarithmic transformation to the target. For the GAM, the logarithmic transformation was part of the model itself (see below). For the ANN, we did not apply this transformation because it did not change the predictive performance in exploratory trainings.

### Predictor selection

In the GAM and the SARIMA models, we selected a subset of predictors. Since the different measures of temperature were highly correlated and the correlation of their average (among the first three lags) with the target was almost equal (mean T = 29.98%, maximum T = 29.58%, minimum T = 28.12%) we used only the mean temperature to avoid high collinearity. In the GAM, the candidate subsets of predictors were given by all possible combinations that had the lag-1 number of cases, and for the other predictors (mean temperature, precipitation and relative humidity), no more than one of the first three lags. For example, the subset {lag-1 number of cases, lag-1 precipitation, lag-3 precipitation} was not considered because it had more than one lag of precipitation. Based on this, the smallest subset had only the lag-1 of the number of cases and the largest had the lag-1 of the number of cases plus one of the first 3 lags of the remaining predictors (mean temperature, precipitation and relative humidity). A total of 64 subsets were tested. For the SARIMA, the approach was the same but without explicitly including the lagged value of the number of cases as a predictor (63 subsets were tested).

### Naïve model

The naïve model was the benchmark and was given by the first lag of the raw time series. Thus, this model predicts the number of cases at time *t* as *t-1*.

### GAM

A GAM is a generalized linear model in which the linear predictor is composed by smooth functions applied to predictors [26]. To select the best GAM, we first selected the subset of predictors that minimized the CV root mean square error (RMSE) on the entire training set. To achieve this, we used a Poisson likelihood and cubic splines with 3 knots on all predictors, for all the tested subsets of predictors. Then, for the best subset of predictors, we trained equivalent models with negative binomial and Gaussian likelihoods. As none of these distributions minimized the CV RMSE, we proceeded with a Poisson likelihood, to tune the type of penalized spline (shrinkage cubic or cubic) and the upper limit on the degrees of freedom (df) associated with the spline (k = df-1 = {3,4,5,6,78}) using the CV RMSE. The general formula of GAM with Poisson likelihood was:

$$y_{i}\sim Poisson(z_{i})$$

$$log(z_{i})=b_{0}+\sum s_{j}(x_{ij}k),$$

where *y*_*i*_ was the observation *i*, *z*_*i*_ is the linear predictor for the observation *i*, *b*_*0*_ is the intercept, *s*_*j*_ is the spline for predictor *x*_*j*_ and *k* is the number of knots.

### ANN

ANN consist of many simple connected processors called neurons or units, linked by directed connections [21,27]. Units are organized in layers and any ANN has at least an input layer activated by data, and an output layer that calculates values used for prediction. ANN can have additional hidden layers between the input and output layers [27]. When a unit is activated, it outputs a value computed from inputs. While units from the input layer get activated by data, units from hidden and output layers get activated by weighted outputs from connected and previously active units [21]. Different structures of units and their connections give rise to different types of ANN. The training of ANN is commonly based on the backpropagation algorithm [28], whereby an optimizer minimizes the loss of an objective function by updating the weights in the opposite direction of the gradient of the objective function. The optimization occurs iteratively over dataset observations, either individually or in batches, and the entire dataset is typically iterated many times (epochs). During training, some units can be randomly dropped out to suppress their contribution to the learning of weights. This technique, knowns as dropout regularization, helps to reduce overfitting [21].

We trained two types of artificial neural networks: the multilayer perceptrons (MLP) and the long-short term recurrent neural networks (LSTM). For both types, the configuration was always as presented in Table 2, with the topologies, predictors and tuned parameters as presented in Table 3. To tune the parameters, we used the CV MSE. To check the convergence, we plotted the loss against the number of epochs.

**Table 2:** Configuration of artificial neural networks.

Epochs	300
Weight initialization	Uniform distribution
Activation of hidden layers	Rectifier linear units
Optimizer	Adaptive moment estimation (Adam)

**Table 3:** Topology and tuned parameters of trained artificial neural networks.

		Topology		Tuned parameters	
Type	Predictors	Hidden layer 1	Hidden layer 2	Batch size	DR
MLP	C	10	5	10, 20, 50	0, 0.2, 0.4
MLP	C, T, P, RH (lags 1–3)	20	10	10, 20, 50	0, 0.2, 0.4
LSTM	C	10	5	10, 20, 50	0, 0.2, 0.4
LSTM	C, T, P, RH (lags 1–3)	20	10	10, 20, 50	0, 0.2, 0.4

MLP: multilayer perceptron, LSTM: long short-term memory recurrent neural networks, C: number of cases, T: temperature (maximum, mean and minimum), P: precipitation, RH: relative humidity, DR: dropout regularization.

### SARIMA

A SARIMA is a linear predictor composed by a non-seasonal autoregressive polynomial (AR) of order *p*, a non-seasonal difference of order *d*, a non-seasonal moving average (MA) of order *q*, a seasonal (AR) of order *P*, a seasonal difference of order D, and a seasonal (MA) of order *Q* [20]. Letting *m* be the number of periods per seasons, SARIMA = (p,d,q)(P,D,Q)_m_. Letting *B = yt/yt-1*, *where* yt and *yt-1* are the number of cases at time *t* and *t-1* respectively, SARIMA for monthly dengue cases can be represented as:

$$(1-\Phi_{1}B-\cdots -\Phi_{p}B^{p})(1-\Phi_{1}B^{12}-\cdots -\Phi_{P}B^{12P}){\left(1-B\right)}^{d}{\left(1-B^{12}\right)}^{D}y_{t}=c+(1-\Theta_{1}B-\cdots -\Theta_{q}B^{q})(1-\Theta_{1}B^{12}-\cdots -\Theta_{Q}B^{12Q})e_{t},$$

where *Φ* and *Θ* are coefficients and *e* is the error.

After a first order non-seasonal differentiation (*d = 1* and *D = 0*), we tested all possible combinations of *p*, *q*, *P* and *Q*, with each of this terms taking a value between 1 and 4, Then we selected the combination with the lowest corrected Akaike’s Information Criterion (AICc) [20].

### Ensemble model

The ensemble model was simply the average prediction of the best GAM, ANN and SARIMA.

### Predictive performance

The RMSE in the test set was the measure of predictive performance for the best GAM, ANN and SARIMA, and the naïve model. The RMSE was defined as:

$$RMSE=\sqrt{(\frac{1}{n}\sum {\left(y_{i}-\hat{y_{i}}\right)}^{2}),}$$

where *n* is the number of observations, *y* is the observed value, and *ŷ* is the predicted value. In addition, these RMSE were divided by the RMSE of the naïve model to obtain a relative measure of performance.

### Software

We used R 3.4.0 and its packages mgcv 1.8–17, forecast 8.0, caret 6.0–76, ggplot2 2.2.1 and gridExtra 2.2.1. We also used Python 3.5.1 and its packages sklearn 0.17.1, pandas 0.19.2 and keras 1.0.6 with Theano as backend.

### Ethics statement

This research was approved by the Comtiê de ética em Pesquisa—Faculdade de Saúde Pública—Universidade de São Paulo. Approval number: 1.687.650. All patient data analyzed were anonymized.

## Results

**Fig 1:**
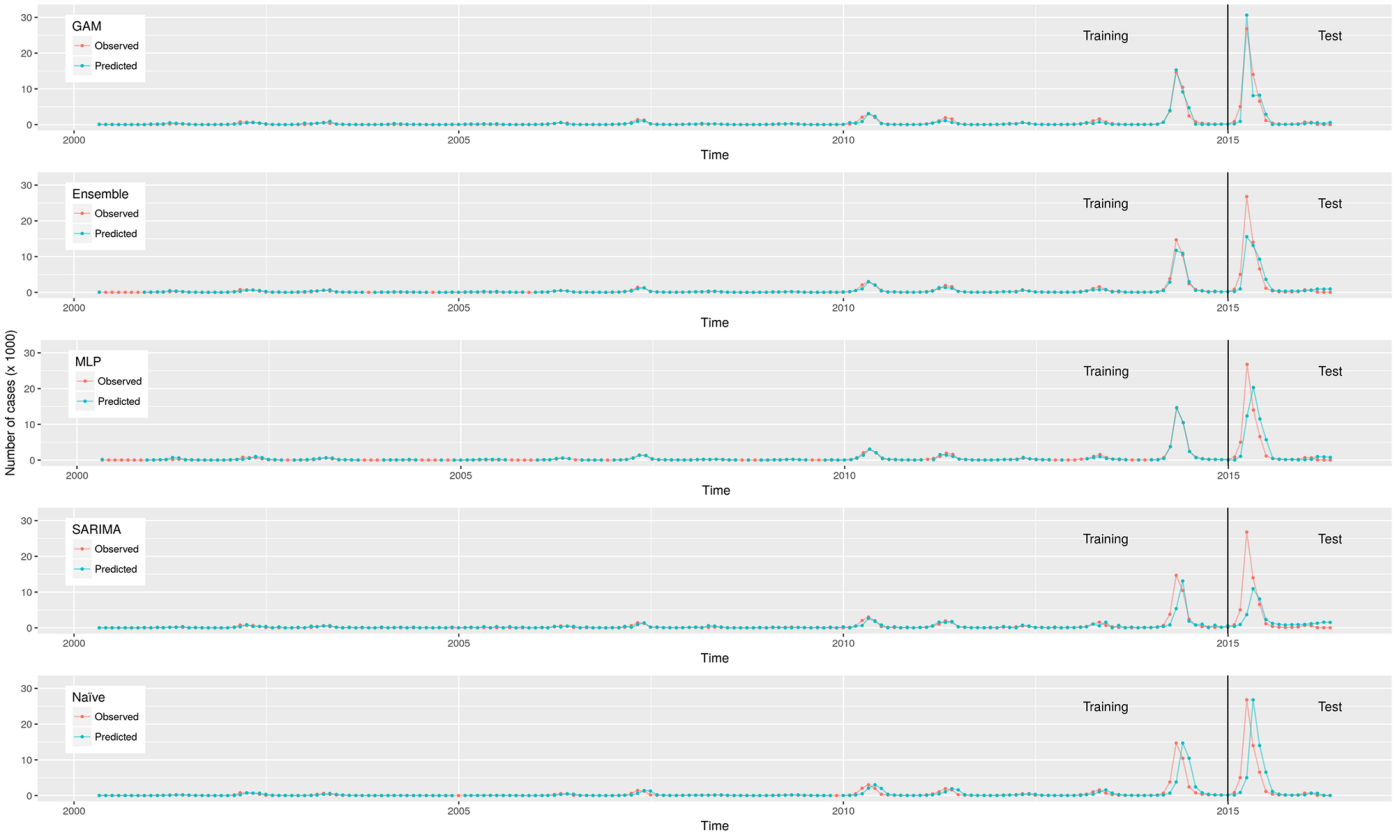
Observed and predicted number of dengue cases in training and test data from São Paulo, Brazil, 2000–2016. Predictions were made by models presented in Table 4.

**Fig 2:**
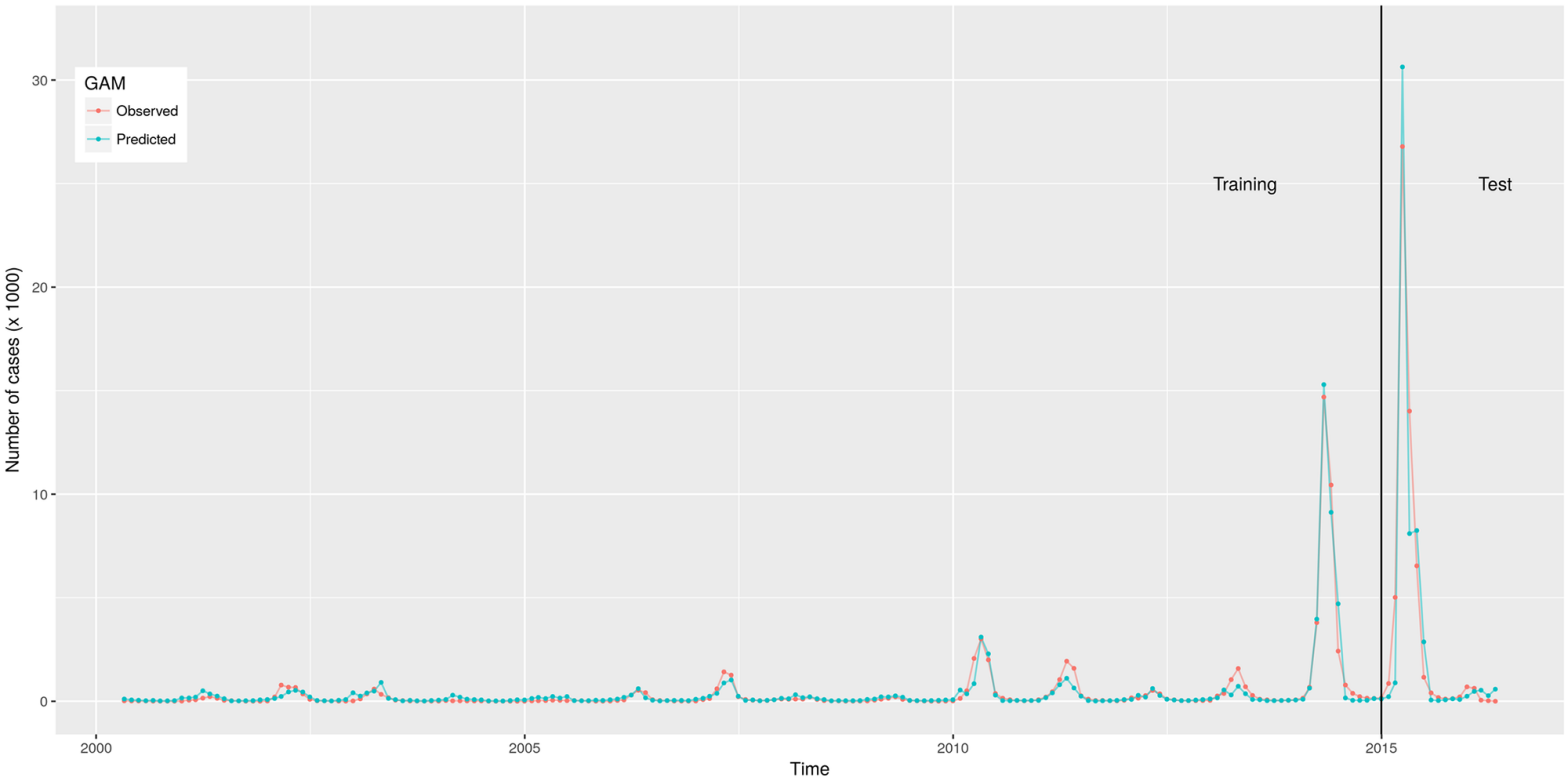
Observed and predicted number of dengue cases in training and test data from São Paulo, Brazil, 2000–2016. Predictions were made by the generalized additive model presented in Table 4.

Table 4 shows the best GAM, ANN, SARIMA and their parametrization. The lowest RMSE in the test set was achieved by the GAM, followed by the ensemble, the MLP, the SARIMA and the naïve model (Table 5) (the LSTM had a RMSE = 5230. It is not included it in Tables 4 and 5 because it was not the best ANN). The GAM correctly predicted large epidemics, especially in 2014 and 2015, which made it more precise (lowest error) than the other models (Figs 1 and 2). According to the GAM, the predicted number of cases increased when any of the predictors increased; in the case of the lagged number of cases, this pattern occurred until it increased approximately 3.5 standard deviations, then, the predicted number of cases decreased slightly (S2 Fig). The number of epochs was enough for the convergence of the MLP (S3 Fig).

**Table 4:** Best generalized additive model (GAM), artificial neural network (ANN) and autoregressive integrated moving average model (SARIMA).

Model	Description	
GAM	Likelihood:	Poisson
	Spline:	Shrinkage cubic
	Knots:	3
	Predictors:	Clag1, Tmaxlag2, Plag1, RHlag1
ANN	Epochs:	300
	Weights initialization:	Uniform distribution
	Activation	Rectifier
	Optimizer:	Adaptive moment estimation
	Type:	Multilayer perceptron
	Units in hidden layer 1:	20
	Units in hidden layer 2:	10
	Batch size:	50
	Dropout regularization:	0
	Predictors:	Clag1-3, Tlag1-3, Plag1-3, RHlag1-3
SARIMA	Transformation:	Natural logarithm
	Non-seasonal autoregressive order:	0
	Non-seasonal difference order:	1
	Non-seasonal moving average order:	3
	Seasonal autoregressive order:	0
	Seasonal difference order:	0
	Seasonal moving average order:	1

C: number of cases, T: temperature (maximum, mean and minimum), Tmax: maximum temperature, P: precipitation and RH: relative humidity.

**Table 5:** Root mean squared errors (RMSE) of predictive models of dengue cases.

Model	RMSE*	RMSE / RMSEnaïve
GAM	2152	0.316
Ensemble	3164	0.465
MLP	4422	0.650
SARIMA	5984	0.879
Naïve	6806	1.000

* Rounded values.

## Discussion

The predictive models captured the seasonal patterns but differed in their capacity to anticipate large epidemics and all outperformed the benchmark in predicting the number of dengue cases one month ahead. Large epidemics occurred between March and May, and in 2014 and 2015, they were markedly different than surrounding months. As a result, the naïve model (benchmark) produced errors on the epidemic peaks and their subsequent month, with a magnitude that preclude its practical use.

Incorporating meteorological variables improved the predictive performance of all models, but the best GAM, SARIMA and ANN differed in the final set of predictors. This difference was in part induced because we did not test all candidate sets in the three types of models owing to their particularities. In the GAM and ANN, it made sense to include lagged values of the number of cases as regressors but in SARIMA, there is no point including this, given the autoregressive and moving average terms. Collinearity may produce strange results in the GAM and SARIMA, and therefore, it is convenient to consider a selection procedure for predictors. On the other hand, the ANN are less sensitive to collinearity because they create linear combinations of the initial inputs to model the target.

The restriction of the potential predictors to the first three lags—the more correlated with the target—was intended to reduce collinearity and overfitting (unnecessary complexity). For the same reason, the GAM and SARIMA were restricted to include at most one of the first three lags of each predictor. We used all the predictors and its first three lags in the ANN but we also tested models with only the first lag of the number of cases to assess the possibility of better predictions with simpler models. Between these two extremes, we could have assessed the combinations considered for the GAM (which are a subset of all possible combinations), however, that would amount to fitting 1152 models (64 combinations of predictors * 9 combinations of tuning parameters * 2 types of ANN) instead of 36 (2 combinations of predictors * 9 combinations of tuning parameters * 2 types of ANN) for cross-validation. Taking into account the delay to collect and prepare the data and the computational cost of fitting thousands of ANN, predictions may take to long to support timely decisions.

Temperature, relative humidity and precipitation composed the set of predictors of the best GAM, SARIMA and ANN, restating the utility of these variables. Our decision to use the mean temperature contrasted with the use of the minimum or maximum temperatures [11,12,29]; however, this is not new [13]. Furthermore, the correlation of each measure of temperature with the target was almost the same.

The best GAM predicted the peaks of 2014 and 2015 better than the other models even when the autocorrelation of its residuals showed that some information were left over. Models for which the maximum number of degrees of freedom was set to more than 2 resulted in uncorrelated errors but did not improve the RMSE neither in the training set nor in the test set.

In the training of the ANN, we chose the adaptive moment estimation optimizer because it is computationally efficient, has minimum memory requirements, works well in practice and compares favorably with other stochastic optimization methods [30]. This optimizer computes adaptive learning rates for each parameter, which allowed us to omit the tuning of the learning rate and the momentum. The rectifier linear units for all layers except the output, was a choice to improve the performance [31]. The output layers did not have activation functions because the aim was to predict untransformed numerical values. The number of epochs was enough to achieve convergence as indicated by the stabilization of the loss across iterations (S2 Fig). The first layer always had more units than predictors, in order to have sufficient flexibility to capture nonlinearities in the data [26]. Since this could lead to overfitting, we tuned the dropout regularization [26].

ANN are typically trained with large datasets, which allow the estimation of large number of parameters. In non systematic search for better models we trained more complex ANN (more layers and more units per layer), which did not improve the predictive performance. Perhaps, the lack of data to build more complex ANN explained the poorer performance relative to the GAM, and perhaps, this also explains the improved performance of the MLP relative to the LSTM.

Regarding the SARIMA, graphical exploration showed that logarithmic transformation stabilized the variability, and nonseasonal differentiation improved the stationarity of the time series. The final model had uncorrelated errors and the Ljung-Box test supported to the conclusion of insignificant remaining autocorrelation. Nonetheless, the peaks of 2014 and 2015 were predicted one month ahead relative to real peaks, resulting in large errors.

The predicted event was the monthly number of cases among residents of São Paulo city. Restricting cases to residents of São Paulo city is not necessarily equivalent to autochthonous cases as some residents may have been infected outside the city while some autochthonous cases may have gone out and notified in other cities. The reason not to have used autochthonous cases was simply the lack of reliable data regarding the probable place of infection. The city of notification would have been an alternative target but official reports are based on resident cases. Furthermore, we decided to build models compatible with existing data.

The GAM had the best performance and included lags of the number of cases and meteorological variables. It predicted epidemics of unprecedented magnitude and can be updated and trained in seconds or few minutes. Furthermore, the meteorological covariates for São Paulo city are updated daily and can be accessed at no charge. This means that the model is suitable for real application to support timely decisions. The model can be trained with data from other cities, but as meteorological stations are not available in every city of São Paulo State, other sources of data must be found.

## Supporting information

S1 Data Data and scripts. (ZIP)

S1 Fig Time series of meteorological predictors. (TIFF)

S2 Fig Nonlinear effects of predictors in the final generalized additive model. (TIFF)

S3 Fig Convergence of the final multilayer perceptron. (TIFF)
