# Supplementary figures and images for: Dengue forecasting in São Paulo city with generalized additive models, artificial neural networks and seasonal autoregressive integrated moving average models
Source: PLoS One. 2018 Apr 2;13(4):e0195065. doi: 10.1371/journal.pone.0195065 (PMC5880372; doi:10.1371/journal.pone.0195065)

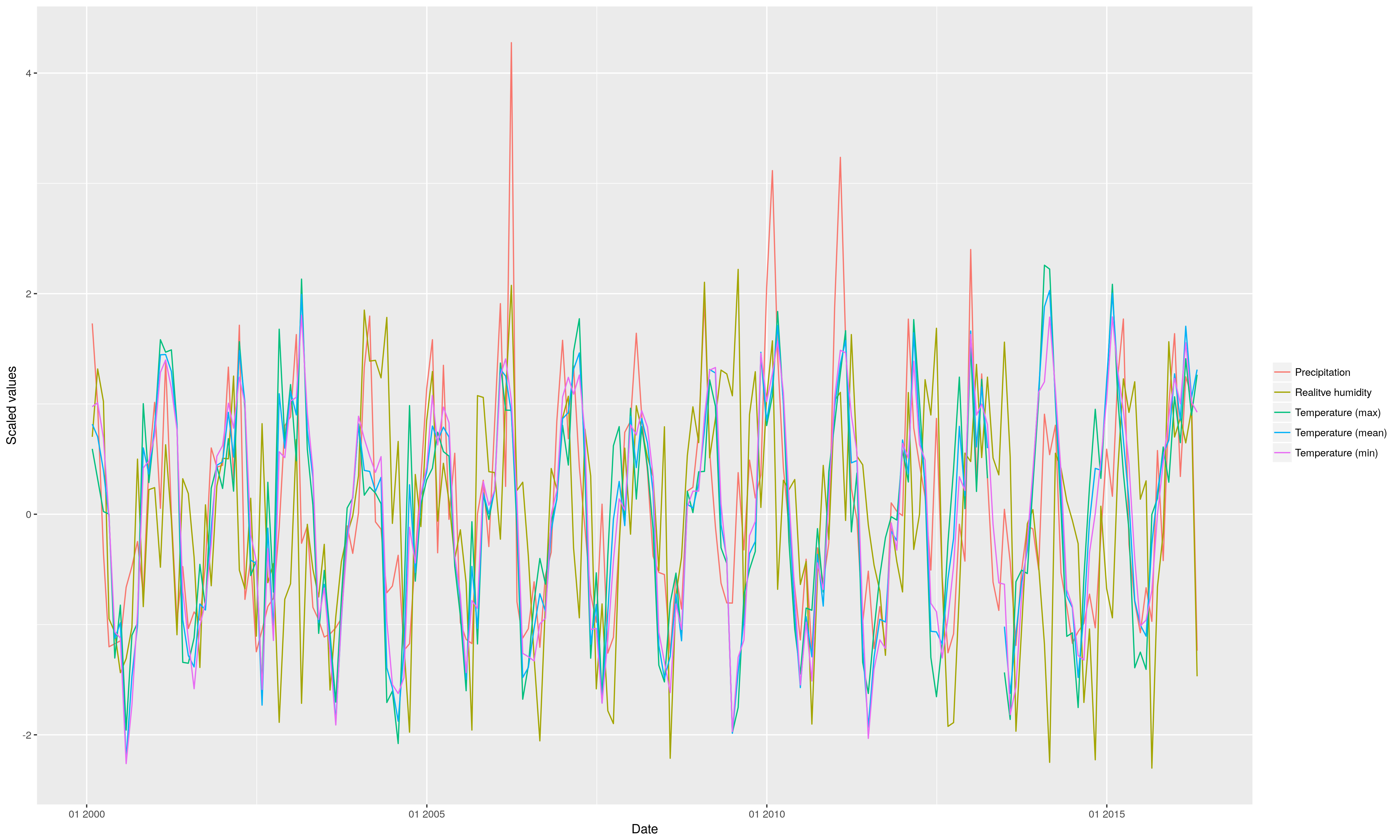

Supplement: S1 Fig — (TIFF) [file pone.0195065.s002.tiff]

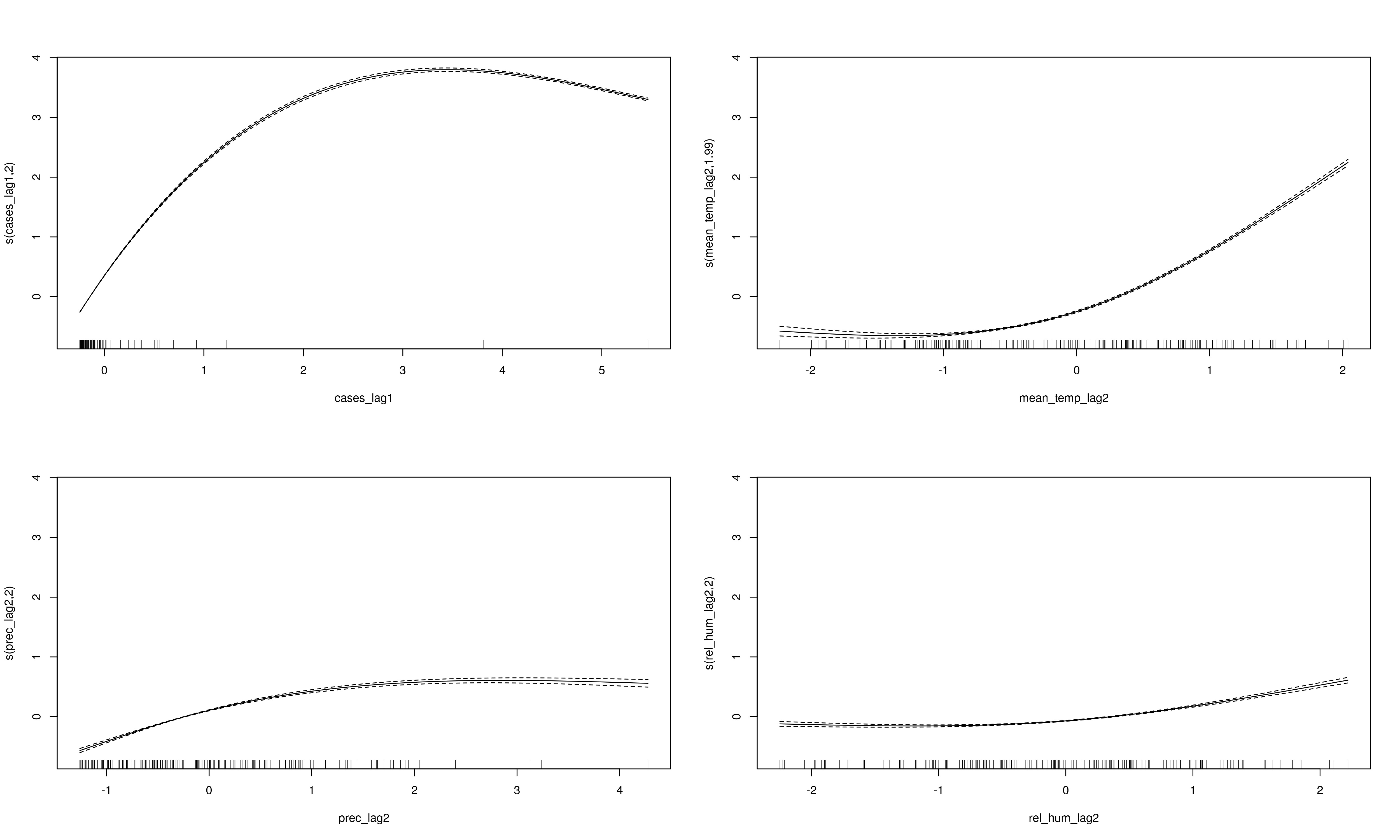

Supplement: S2 Fig — (TIFF) [file pone.0195065.s003.tiff]

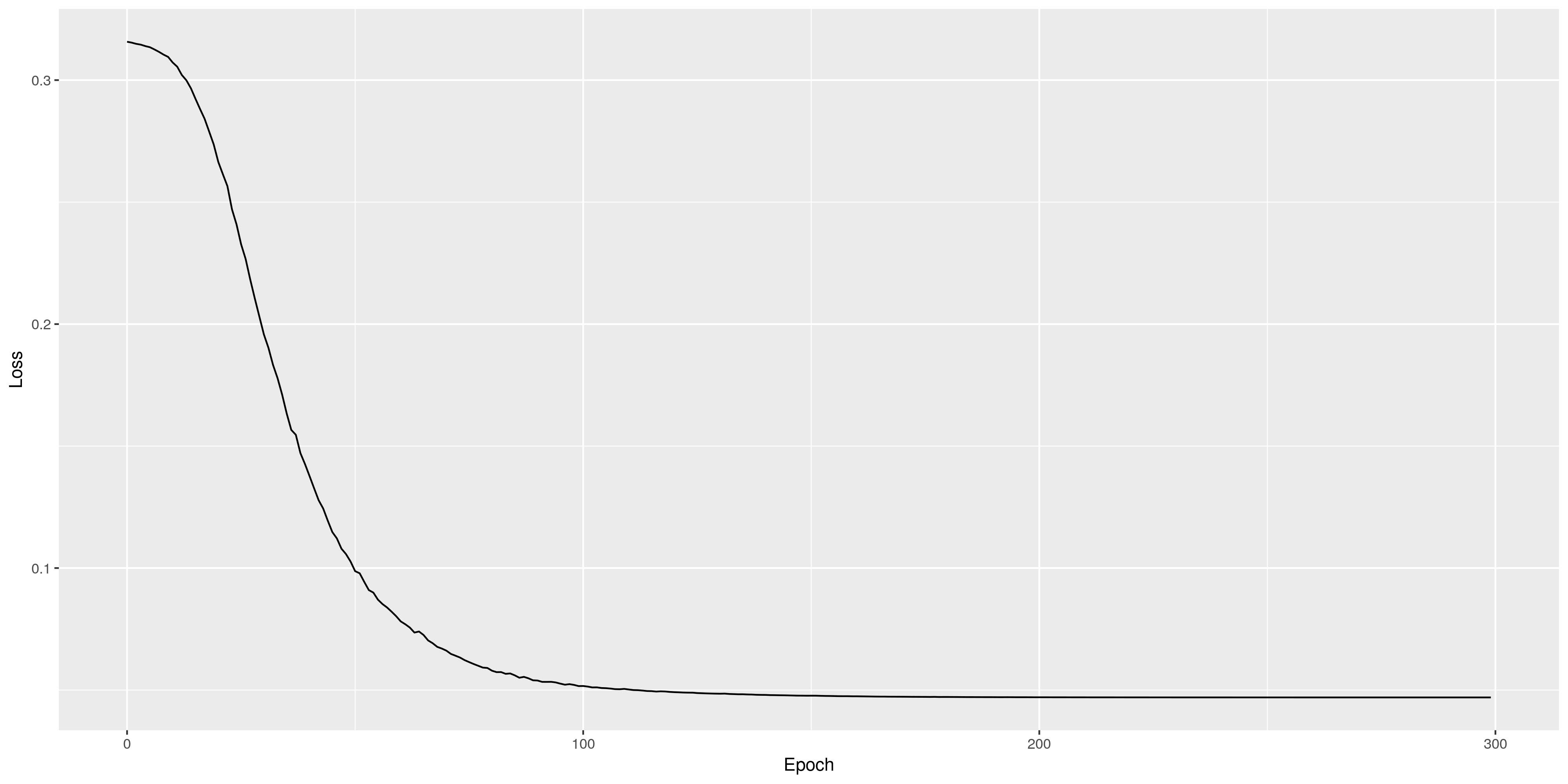

Supplement: S3 Fig — (TIFF) [file pone.0195065.s004.tiff]
